# The Evolution of AIFA Registries to Support Managed Entry Agreements for Orphan Medicinal Products in Italy

**DOI:** 10.3389/fphar.2021.699466

**Published:** 2021-08-10

**Authors:** Entela Xoxi, Karen M Facey, Americo Cicchetti

**Affiliations:** ^1^ Graduate School of Health Economics and Management, Catholic University of the Sacred Heart, Rome, Italy; ^2^ Usher Institute, University of Edinburgh, Edinburgh, United Kingdom

**Keywords:** registries, managed entry agreement, orphan medicinal product, rare disease treatment, regulatory approval, innovativeness recognition, outcome-based managed entry agreements

## Abstract

Italy has a well-established prominent system of national registries to support managed entry agreements (MEAs), monitoring innovative medicinal products (MPs) with clinical as well as economic uncertainties to ensure appropriate use and best value for money. The technological architecture of the registries is funded by pharmaceutical companies, but fully governed by the national medicines agency (AIFA). A desktop analysis was undertaken of data over a 15-year timeframe of all AIFA indication-based registries and associated EMA information. The characteristics of registries were evaluated, comparing orphan MPs vs. all MPs exploring cancer and non-cancer indications. OMP (orphan medicinal product) registries’ type vs. AIFA innovation status and EMA approval was reviewed. Of the 283 registries, 182 are appropriateness registries (35.2% relate to OMPs, with an almost equal split of cancer vs. non-cancer for OMPs and MPs), 35 include financial-based agreements [20% OMPs (2 non-cancer, 5 cancer)], and 60 registries are payment by result agreements [23.3% OMPs (4 non-cancer, 10 cancer)]. Most OMPs (53/88) came through the normal regulatory route. With the strengthening of the system for evaluation of innovation, fewer outcomes-based registries have been instigated. AIFA has overcome many of the challenges experienced with MEA through developing an integrated national web-based data collection system: the challenge that remains for AIFA is to move from using the system for individual patient decisions about treatment to reviewing the wealth of data it now holds to optimize healthcare.

## Background

### The Need for Managed Entry Agreements With Orphan Medicinal Products

It is recognized that there are high unmet needs for treatment for rare diseases (defined in Europe as a condition that occurs in less than 1 in 2,000 people). As a result, the European Medicines Agency (EMA) established a special regulatory process to support the development and approval of treatments that are expected to have significant benefit for rare diseases, designating them as orphan medicinal products (OMPs).

The heterogeneous nature and limited clinical understanding about many rare diseases combined with a small number of patients to treat, who are sparsely located, means that clinical development of these products can be challenging (Facey et al., 2014). Furthermore, these products often come with a high price due to the complexities of drug development and the highly innovative medicines needed to treat these conditions, and this is particularly the case for ultra-rare diseases (Berdud et al., 2020), which make up the majority of rare diseases (Nguengang Wakap et al., 2020). This gives healthcare payers major challenges when they need to determine the added value of such medicines and make reimbursement/appraisal decisions based on incomplete evidence with major gaps and clinical uncertainties. Some countries have adapted their appraisal and decision-making processes to allow flexibility for OMPs and novel reimbursement agreements (Morel et al., 2013; Wenzl and Chapman, 2019). These managed entry agreements (MEAs) or risk-sharing agreements (RSAs) enable access to treatments where traditional appraisal processes would not lead to their use/reimbursement.

Klemp et al. (2011) explain that an MEA is an arrangement between a company and a payer/provider that enables access to (reimbursement of) a product subject to specific conditions to manage budget impact, optimize performance, or address uncertainty relating to clinical and/or cost-effectiveness. These schemes can range from simple discounts to the price that is applied to all patients, through to complex real-world research studies where data is collected to address uncertainties and inform a reappraisal or renegotiation of price. The authors note the challenges associated with schemes where data collection is required, and a number of countries have reported failure of such schemes to collect the required data and inform reimbursement renegotiations. (Garrison et al., 2013; Facey et al., 2014; Faulkner et al., 2016).

Challenges with MEA include agreement on funding (of treatment and data collection), data collection (what to collect in a timely manner and how to ensure quality and completeness), confidentiality issues, and use of data to inform subsequent decisions.

As a result, over the past decade, use of administratively simple financial-based schemes has become a commonplace with application of a confidential discount to the published list price. However, for high price OMPs, with major uncertainties in the clinical evidence base, this is not sufficient, and there is interest in the potential of MEA that can help determine/check the real-world effectiveness of OMPs within the healthcare setting. Such schemes may be termed as outcomes-based MEA (OBMEA) or performance-based RSA.

OBMEA occur at two levels (individual and/or population) and are likely to be combined with a financial agreement such as a confidential discount:

• Individual—ensuring appropriate use and assessment of outcomes for each patient (paying only if response achieved or refund if response not achieved, continuation of treatment according to certain responses);
• Population—collection of data to aggregate for reappraisal.

Italy has been internationally recognized as establishing a system to implement financial-based MEA and individual OBMEA in order to monitor high-price medicinal products (MP) through a national web-based registry system funded by pharmaceutical companies, but governed by the health system (Xoxi et al., 2012; Montilla et al., 2015; Cicchetti et al., 2017).

Work Package 10 (WP10) of the EC H2020 funded the IMPACT-HTA project which is developing guidance on novel approaches to appraise OMPs, including the use and implementation of OBMEA. IMPACT HTA WP10 undertook several streams of research to evaluate issues related to the implementation of OBMEA. This began with a review of the literature related to implementation of population-based OBMEA, identifying issues related to initiation of OBMEA, and conduct and reappraisal. Multistakeholder workshops were held with all those involved in the OBMEA used by the National Institute for Health and Care Excellence in its highly specialized technologies program (for ultra-rare diseases) to explore issues of implementing population-based OBMEA. This gave an example of an OBMEA system that was accessing the health data in a bespoke fashion for each product from various sources. Alongside this, the research presented here was undertaken to explore a very different system, using a national registry system on a standard platform for individual-based OBMEA. The objective of this research was to explore the history to the establishment of the AIFA registry system, how its use had evolved over the years, and lessons learned.

### Development of registries in Italian Medicine Agency

The Italian Medicine Agency (Agenzia Italiana del Farmaco, AIFA) is the national authority responsible for the regulatory, pricing and reimbursement (PR), and HTA activities related to pharmaceuticals including governance of pharmaceutical expenditure (AIFA, 2003). It is supported by two advisory committees: Technical-Scientific (*Commissione Tecnico-Scientifico*, CTS) and pricing and reimbursement (*Comitato Prezzo e Rimborso*, CPR). Registries are an important operational part of the PR negotiation between AIFA and the pharmaceutical industry constituting a key element of the contract between the two parties.

OMPs are reimbursed by the Italian NHS in accordance with the national regulation. AIFA gives OMP-PR dossiers (together with those concerning medicines of exceptional therapeutic relevance) a priority over other pending applications. In such cases, the assessment period is reduced from 180 days (standard) to 100 days (so-called fast-track authorization). In addition, AIFA has different regulations to enable early patient access to OMPs. One of the most used is based on Law n. 648/1996 (Gazzetta Ufficiale, 1996) that allows some OMPs to be reimbursed in the NHS before marketing authorization. This applies to OMPs for severe conditions which have positive results from phase II clinical trials and no alternative therapeutic option or one that is too expensive according to the n. 79/2014 law (Gazzetta Ufficiale, 2014b). The inclusion of a MP in the so-called 648 list is enacted by AIFA on the basis of a documented request from patients’ associations, scientific societies, health facilities, universities, and following recommendations from CTS. Within this, there is a provision to enable AIFA to undertake early access monitoring with a “648/96-registry” (648/96-R) before EMA has approved the marketing authorization. Once EMA approval has been obtained, the 648/96-R could be transformed into a standard registry (R) in accordance with the therapeutic indication negotiated by AIFA.

AIFA registries are not observational studies but prospective administrative data from clinical practice for MPs to be used mainly in the hospital setting (H classification) as a duplication of hospital health data flows, and this characterization and other elements described in this article contains the strongest criticality of the AIFA registries: that of remaining disconnected from other national/local data sources because being built from scratch by the agency with a clear purpose of appropriateness’ verification as part of PR contract between AIFA and pharmaceutical companies. Originally established for cancer products, in the period 2005–2007, the use of “monitoring registries” has been expanded to many therapeutic areas (neurology, endocrinology, and cardiology) where it was deemed necessary by AIFA advisory committees that verification of appropriate use and precise monitoring of NHS expenditure was needed. The principles for establishment of registries were: 1) well-defined therapeutic indication for the specified disease areas in the treatment phase; 2) defined clinical uncertainties (particularly for products that had undergone any form of accelerated regulatory approval); and 3) economic impact of the MP on the NHS.

It should be added that AIFA also monitors a few so-called A-classifications MP (such as oral anticoagulants) in a more simplified way through national web-based therapeutic plans which are built on the same platform as the registries: these are not the subject of our analysis.

Based on last update of National Report of Medicines Use in Italy 2020 (AIFA, 2020a), both types of AIFA data collection enrolled 2,285,899 patients on treatment.

Law 135/2012 (Gazzetta Ufficiale, 2012) recognized registries as an integral part of the NHS Information System, while the additional regulations that were introduced [125/2015 (Gazzetta Ufficiale, 2015b), 232/2016 (Gazzetta Ufficiale, 2016), and 205/2017 (Gazzetta Ufficiale, 2017d)] attributed specific responsibilities for drug-assessment for PR renegotiation purposes.

The administrative flow for the establishment of a registry starts from the CTS (Technical-Scientific Commission). For each MP/indication, the CTS determines the place in therapy, reimbursement class, innovation status, and assesses the uncertainties. CTS issues a mandate to the AIFA registries office for the development of a registry and if an OBMEA is to be enacted, clinical experts (in accordance with the conflict of interest policy) and the pharmaceutical company will be involved in the establishment. The flow can take months between the parties involved in the discussions: the final result is agreement on the eligibility criteria for use of the product on the NHS, the clinical data to be collected, and any OBMEA to be implemented. To complete the process, the CTS opinion then transfers to CPR for price negotiation and discussion of any other agreements such as those relating to financial issues. The procedure ends after approval by the AIFA Board of Directors with the publication of the AIFA Deliberation in the Official Journal. Eventual changes (so-called versioning) based on clinical practice experience or regulatory decisions are a regular part of the lifecycle of a registry and MEA.

### Determination of Innovation Status

The assessment of innovation status of a MP is an essential part of the reimbursement determination in Italy. Before 2017, AIFA used an *ad hoc* algorithm (AIFA, 2007) which was then changed in 2017 to a more transparent and a systematic value framework (Gazzetta Ufficiale, 2017c; Gazzetta Ufficiale, 2017e). This new approach related to the current legislation (Gazzetta Ufficiale, 2016), which specifies that, for fully innovative products, AIFA registries are mandatory in order to manage pharmaceutical governance and related uncertainties (clinical and/or financial). The new approach is based on three criteria: 1) unmet need, 2) clinical added value, and 3) robustness of clinical evidence. For the first two criteria AIFA assign, at a therapeutic indication-level, a five-points score (maximum, important, moderate, low/poor, and absent) plus a four-point GRADE (GRADE Working Group, 2004) score (high, moderate, low, and very low). Generally, a MP which has been recognized as having the first two criteria at the maximum or an important score and a high quality of evidence may be considered innovative. Intermediate situations are evaluated on a case by case basis, considering the relative weight of the individual criteria.

For OMPs, the CTS takes into account the difficulty of conducting randomized controlled trials: in these cases, if the therapeutic need is high and the indications on added value are strong, the CTS might accept low quality of evidence. At the end of the process, the CTS publishes a brief report (Innovation Assessment Report) (AIFA, 2020g) which could have three final judgments in terms of recognition of innovation: 1) fully innovative, 2) conditionally or potentially innovative, and 3) non-innovative. The “fully innovative” status is accompanied by inclusion in one of two funds (each of 500 million euros for cancer and other innovative MPs) and mandatory inclusion in the regional therapeutic formularies (as well as the “conditionally or potentially innovative” MPs). As established by the 2017 budget law (Gazzetta Ufficiale, 2016), the recognition of innovation and the consequent benefits have a maximum duration of 36 months (also valid for the first in class). The permanence of the innovation status attributed to a MP will be reconsidered if there is evidence that justifies its reevaluation. In any case, for “conditionally or potentially innovative” products a reevaluation at least 18 months from its grant is mandatory, and the availability of new evidence that was positively assessed by the CTS may lead to a “fully innovative” status, with the conferment of benefits for the remaining time.

## Objective

The objective of this research was to review how the AIFA system for registries to support MEA has been developed and evolved, focusing on any differences for OMPs. As many aspects impact the decision about the form of an MEA, this analysis has explored whether there is any association between important policy factors and the form of the registry. The type of AIFA registries, their duration, the form of MEA they support, and how they differ for OMPs vs. other MPs, for cancer and non-cancer indications, has been investigated. For OMPs, association with regulatory approval and innovation status is then considered.

## Methods

### Data Extraction

**FIGURE 1:**
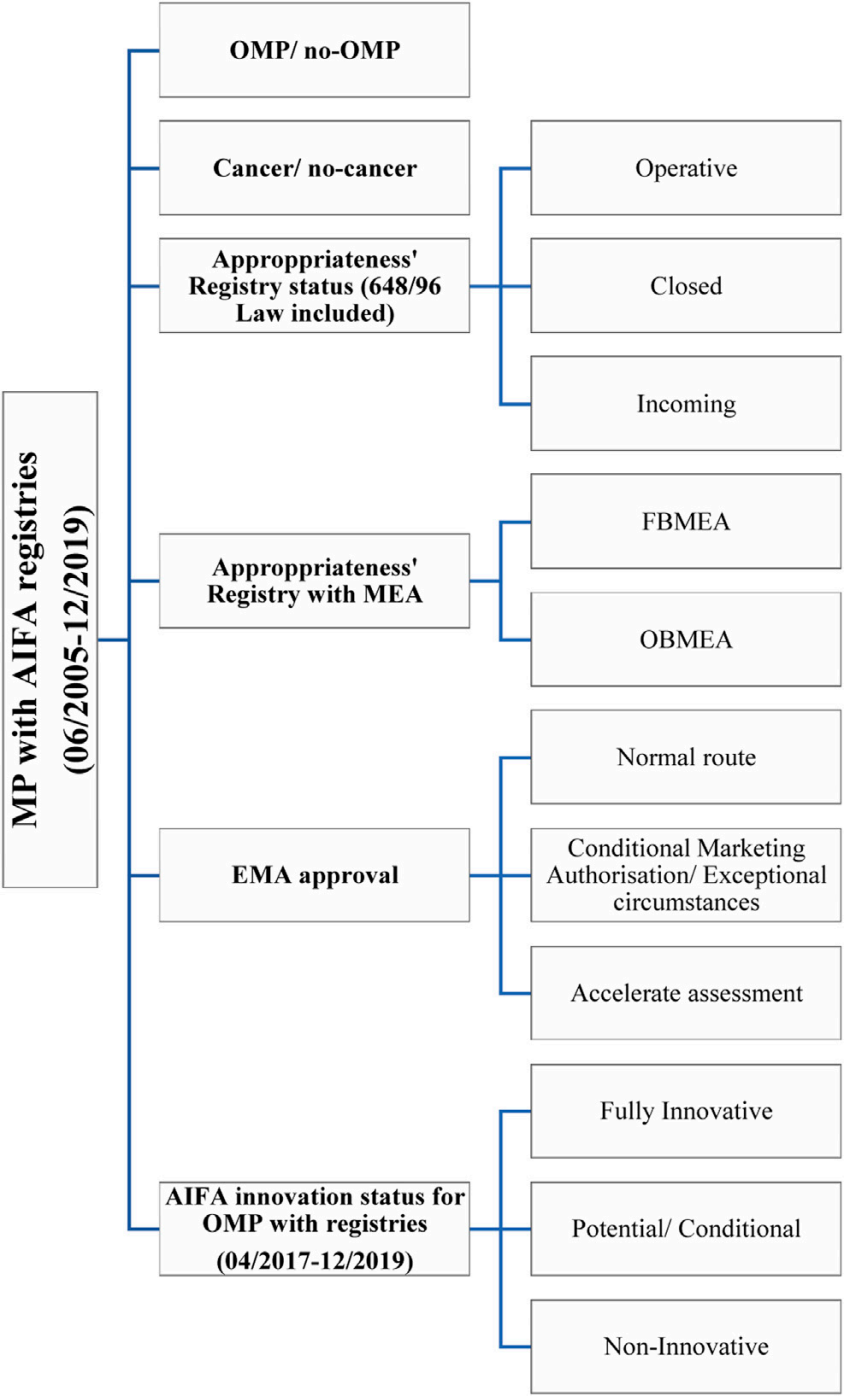
The desktop analysis scheme. MP, medicinal product; OMP, orphan medicinal product.

A desktop analysis (Figure 1) was performed on publicly available information in English and Italian extracting information from:

• AIFA registries webpages (AIFA, 2020; AIFA, 2020c) including details for each MP of therapeutic indication, data collection, termed here as registry (R), inclusion and exclusion criteria (link to pdf format registry e-forms), starting and end date of registry with or without managed entry agreement, status of registry or MEA (operative, closed, or incoming), and version of registry. The extraction was carried out for registries between June 19, 2005 (the launch of the first registry), and December 31, 2019, and consisted of .xlsx format file downloads.
• EMA European public assessment report (EPAR) (EMA, 2019) download medicine data including information on orphan designation [OD (EMA, 2020d)], approval procedures [accelerated assessment (AA (EMA, 2020)], conditional marketing authorization [CMA (EMA, 2020a)] or exceptional circumstances [EC (EMA, 2020b)], marketing authorization date, therapeutic indications, market authorization holder (MAH), and summary of product characteristics (SmPC).
• AIFA innovativeness’ recognition (AIFA, 2020g) was made publicly available on AIFA’s website starting from January 2018 covering the period from April 2017 to December 2019. The information extracted contains the final CTS judgment on “fully innovative,” “conditionally or potentially innovative,” or “non-innovative” assessed with the criteria of 2017 (Gazzetta Ufficiale, 2016; Gazzetta Ufficiale, 2017c; Gazzetta Ufficiale, 2017e).

As some MPs have several therapeutic indications and the innovativeness criteria may differ by indication, all analyses were based on therapeutic indication, not just MP. The characteristics of registries (Montilla et al., 2015; Cicchetti et al., 2017) established over the 15-year timeframe of our analysis were evaluated, comparing OMPs vs. all MPs. As the rare disease challenges identified in the introduction are not so apparent for rare cancers (due to better clinical expertise and infrastructure to support good clinical trials), cancer and non-cancer indications were analyzed. The type of registries vs. AIFA innovation status and form of EMA regulatory approval was also explored.

## Results

Results of our analysis will be presented in four different sections. In the first section, the evolution of registries and their establishment in the last 15 years will be presented comparing trends in OMPs with other MPs. A second section will explore the characteristics of MEAs associated with registries both for OMPs and MPs. The third section will the focus on the correlation between the form of approval by EMA and the nature of the registries implemented by AIFA. Finally, an analysis of the implication for registries opening due to the declaration of innovativeness will be presented.

### AIFA Registries Trend and Timeline

**FIGURE 2:**
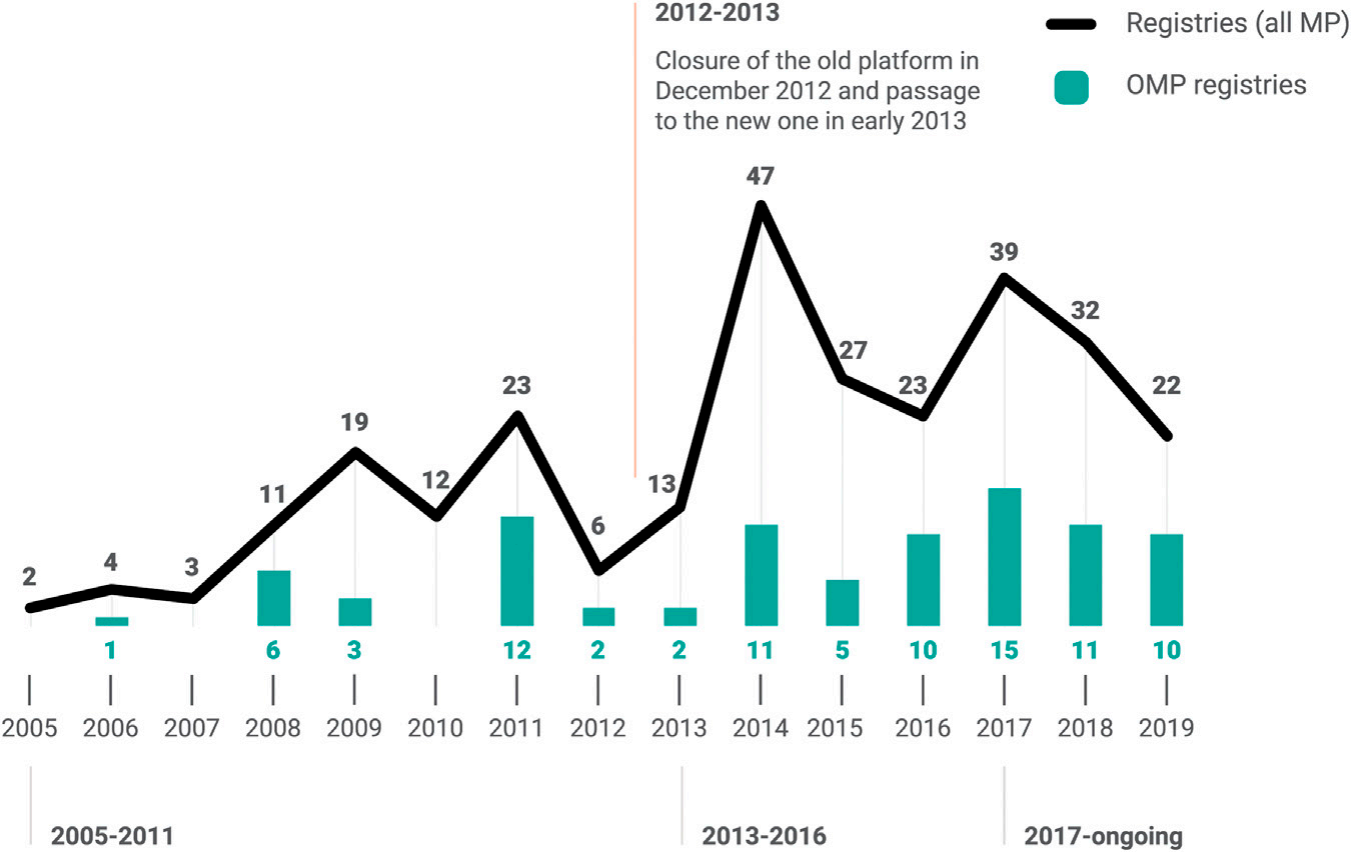
The incidence of AIFA registries. MP, medicinal product; OMP, orphan medicinal product.

Figure 2 presents the number of new registries each year from the inception of registries in 2005, highlighting those for OMPs in green. Up to the end of 2019, there were a total of 283 (indication-based) registries for 159 MPs. Of these 88 (31%) were orphan indications, relating to 52 OMPs. There were 259 registries standard (R) and 24 in early access (648/96-R) with 198 active, 77 closed (65 R and 12,648/96-R), and 8 in paper monitoring.

To determine the duration of registries for OMPs, the analysis identified the 77 closed registries, 11 of which are for OMPs. In some cases, the registry was not definitively closed but there was a switch from an early access registry (648/96-R) into a standard registry [for example Adcetris® (AIFA, 2012; Gazzetta Ufficiale, 2014a) and Iclusig® (AIFA, 2014; Gazzetta Ufficiale, 2015)]. Seven OMPs, were withdrawn from use in EU, of which two were advanced therapeutic medicinal products (ATMP) [Lartruvo® (Gazzetta Ufficiale, 2017a; AIFA, 2019e; EMA, 2019a; EMA, 2020c) and Zalmoxis® (Gazzetta Ufficiale, 2018a; AIFA, 2019b; EMA, 2019b)]. Excluding these, there are two relevant closed registries for OMPs that allow determination of their duration: 12 years for Nexavar® (Gazzetta Ufficiale, 2006; AIFA, 2018e) for renal cell carcinoma indication and 9 years for Kuvan® (Gazzetta Ufficiale, 2009; AIFA, 2017d) for the treatment of hyperphenylalaninemia.

### Type of Agenzia Italiana del Farmaco Managaed Entry Agreements

Different types of MEA (Xoxi et al., 2012; Montilla et al., 2015; Cicchetti et al., 2017) have been implemented within the AIFA registry system since 2005. Table 1 presents the form of MEA for each of the 283 therapeutic indications in this analysis. Of the 283 indication-based registries in our analysis, 182 are only for appropriateness, 35 have an additional financial-based agreement (FbA), and 60 uses PbR. The remaining six are for two forms of schemes that have been rarely used to date [two RS (Gazzetta Ufficiale, 2005a; Gazzetta Ufficiale, 2008a) and one combination PbR with FbA (Gazzetta Ufficiale, 2013a)].

**TABLE 1:** MEA classification implemented in AIFA appropriateness' registries (2005–2019).

Managed Entry Agreement	Definition	Medicinal product (non-OMP)	OMP
Appropriateness’ verification only (A)	Verification of use according to authorised indication, including AIFA restrictions. **It is applied to all the registries**.	ALECENSA, ALIMTA, AVASTIN, BAVENCIO, BENLYSTA, CABOMETYX, CIMZIA, DUPIXENT, EMPLICITI, ENBREL, ENTYVIO, EPCLUSA, EXVIERA, EYLEA, GAMTEN, GILENYA, HARVONI, HEMLIBRA, HUMIRA, IBRANCE, IGVENA, ILARIS, IMFINZI, INFLECTRA, JAKAVI, JETREA, JEVTANA, JINARC, KEYTRUDA, KINERET, KISQALI, LEMTRADA, LENVIMA, LUCENTIS, LYNPARZA, MABTHERA, MEPACT, NPLATE, OCTAGAM, ODOMZO, OPDIVO, ORKAMBI, PERJETA, PIXUVRI, PRALUENT, PRIVIGEN, REMICADE, REMOVAB, REPATHA, ROACTEMRA, RUBRACA, SAMSCA, SIMPONI, SOVALDI, STIVARGA, TAFINLAR, TECENTRIQ, TYSABRI, VELCADE, VENCLYXTO, VERZENIOS, VOSEVI, XALKORI, XOFIGO, XOLAIR, XTANDI, YERVOY, ZEVALIN, ZINBRYTA, ZYDELIG, ZYKADIA, ZYTIGA	ADCETRIS, ADEMPAS, ATRIANCE, BESPONSA, COMETRIQ, DARZALEX, DELTYBA, ELAPRASE, ESBRIET, FARYDAK, GAZYVARO, IMBRUVICA, IMNOVID, KALYDECO, KANUMA, KUVAN, MYLOTARG, NINLARO, OFEV, ORFADIN, OXERVATE, PREVYMIS, QARZIBA, RAXONE, REVLIMID, REVOLADE, RYDAPT, SOLIRIS, SPINRAZA, THALIDOMIDE CELGENE, TRISENOX, VYNDAQEL, VYXEOS, ZALMOXIS, ZEJULA
Financial-based [Cost-sharing (CS) & Capping (capp)]	Capp agreed total budget cap, eligible patients treated for free after cap reached. CS is the application of a discount (fixed or variable, from MAH to NHS) on the cost of the treatment cycles/months for all eligible patients.	ALECENSA, ARZERRA, AVASTIN, CAPRELSA, CYRAMZA, DAKLINZA, ERIVEDGE, ILARIS, MAVIRET, OLYSIO, OPDIVO, SPRYCEL, SUTENT, TAGRISSO, TARCEVA, TORISEL, VELCADE, VIDAZA, VIEKIRAX, VOTRIENT, ZALTRAP, ZEPATIER	CRYSVITA, DACOGEN, KYPROLIS, LARTRUVO, NEXAVAR, SIRTURO, TASIGNA
Risk-sharing (RS)	Discount (fixed, usually 50% from MAH to NHS) for non-responders (according to pre-defined criterion)	VECTIBIX, ERBITUX	
Payment BY result (PbR)	Extends the modalities of the RS by providing for 100% reimbursement by the MAH to NHS for non-responders. It consists of a months-based payback model.	ABRAXANE, AFINITOR, ALIMTA, AVASTIN, BOSULIF, COTELLIC, ERBITUX, GIOTRIF, HALAVEN, HERCEPTIN, INLYTA, IRESSA, JAVLOR, KADCYLA, MACUGEN, SUTENT, TORISEL, TYVERB, VARGATEF, VECTIBIX, VOTRIENT, XALKORI, XIAPEX, YERVOY, ZELBORAF	ADCETRIS, BLINCYTO, HOLOCLAR, ICLUSIG, MOZOBIL, NEXAVAR, SIGNIFOR, STRIMVELIS, TASIGNA, YONDELIS
Combo PbR and CS		SATIVEX	
Success fee (SF)	It is based on the definition of the responder: the hospital/ pharmacy pays the MAH only if the treatment is successful after starting it with free supply.	NA	NA
Payment AT result (PaR)	Exploits the SF paradigm: the hospital pays the MAH only if the treatment is successful (outcome-based) after starting with free supply or an up-front payment. It involves an annual **payment model**.		KYMRIAH, YESCARTA

Given a medicinal product can have different MEA due to multiple therapeutic indications and also given the MEA dynamism, we limit our representation to only capture the forms of MEA for a MP/OMP without entering into the details for each therapeutic indication/line of treatments and current status of MEA/registry.

MAH, marketing authorisation holder; OMP, orphan medicinal product.

One of the fundamental characteristics of AIFA registries is that they all enable collection of appropriateness data to verify use according to the authorized indication (label) and avoid off-label use. In many cases, these registries are augmented to include other forms of MEA (financial or outcomes-based).

**FIGURE 3:**
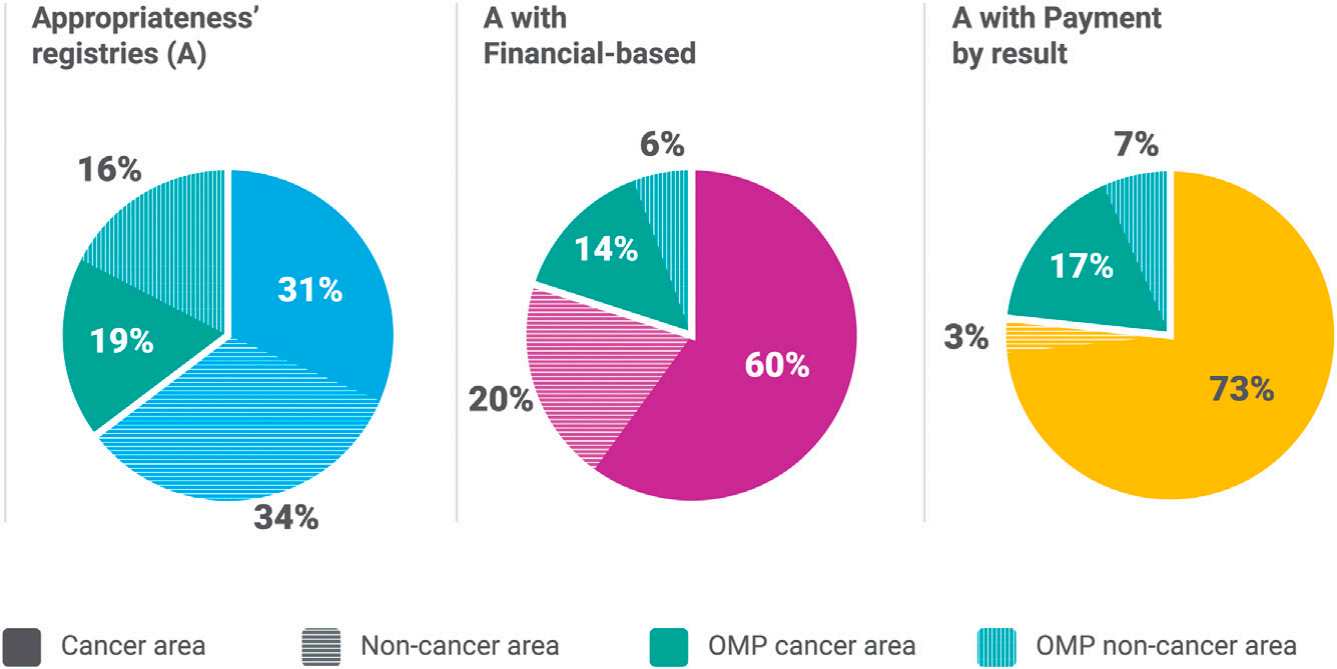
AIFA MEAs distribution in cancer (solid color) and non-cancer indications (color with pattern).

Figure 3 presents the form of MEA associated with the registries by cancer and OMP. This analysis shows that 62% of the registries are related to oncology products, which is interesting given that the registries started in 2005 for a cancer MP [trastuzumab (Gazzetta Ufficiale, 2005) for the early stage adjuvant therapy in the treatment of HER-2 positive breast cancer].

For the 182 appropriateness only registries, 35.2% (64) relate to OMPs, and there is an almost equal split of cancer vs. non-cancer for both the OMPs and MPs. For the 35 registries with financial-based agreements, 20% (7) were OMPs (2 non-cancer, 5 cancer). For 60 registries with payment by result agreements 23.3% are for OMPs (4 non-cancer and 10 cancer).

SF was first proposed in 2013 [pirfenidone (Gazzetta Ufficiale, 2013) case for the treatment of mild to moderate idiopathic pulmonary fibrosis] and again in 2015 [pomalidomide (Gazzetta Ufficiale, 2015a) for myeloma multiple treatment]. However, these two SF agreements were never implemented, but converted (AIFA, 2015; AIFA, 2018c) to two appropriateness registries. The reason seems to be linked with some administrative issues. Unfortunately, we did not find any explanation on it.

**FIGURE 4:**
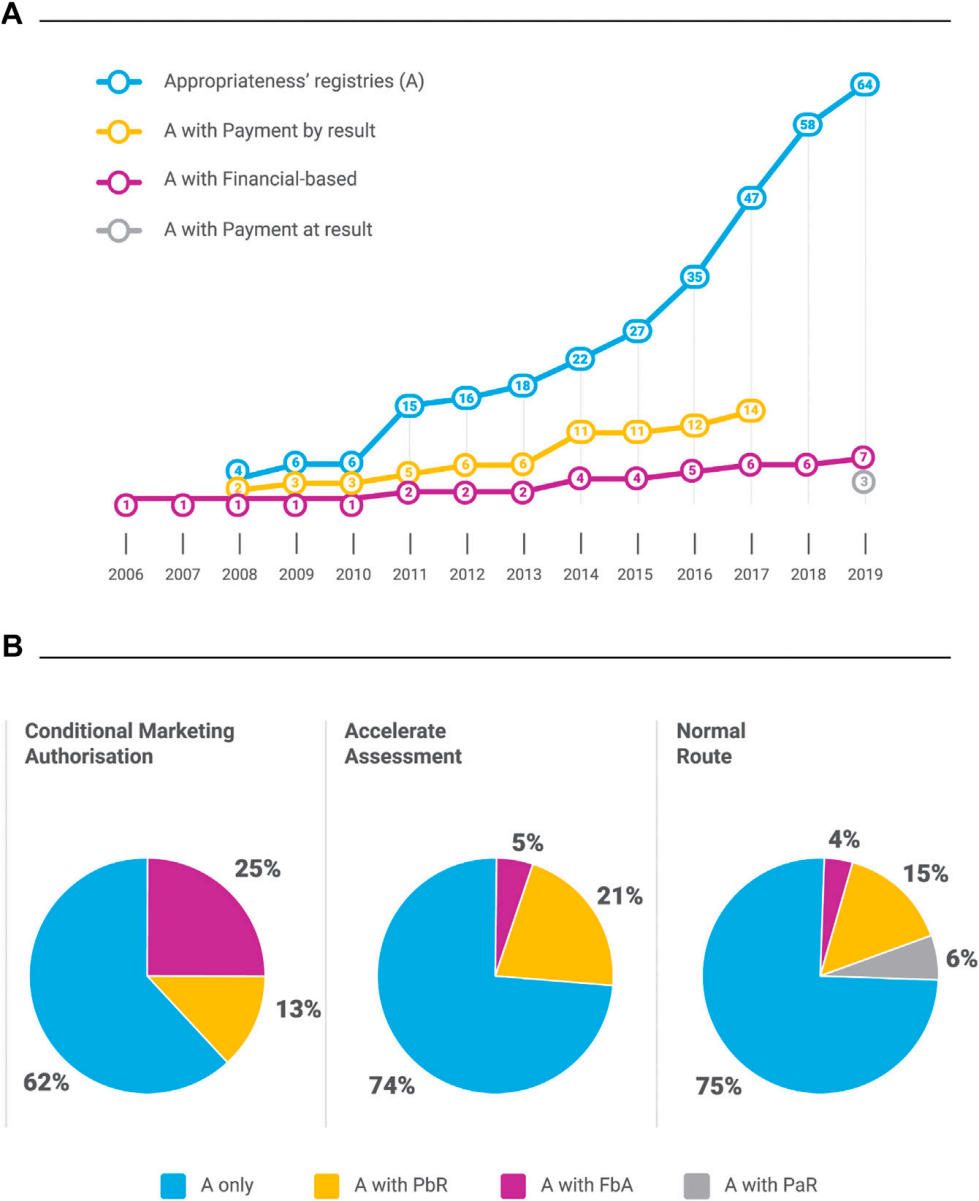
OMP registries and EMA regulatory pathways. A, appropriateness; OMP, orphan medicinal product; PbR, payment by result; FbA, fincial-based agreement; PaR, payment at result.

The three (indication-based) PaR MEA are a new form of payment for the two CAR-T cell therapies (AIFA, 2019; AIFA, 2019a) (as reported in Figure 4A and Table 2) implemented in 2019.

**TABLE 2:** OMPs registries and innovation status (2017–2019 analysis).

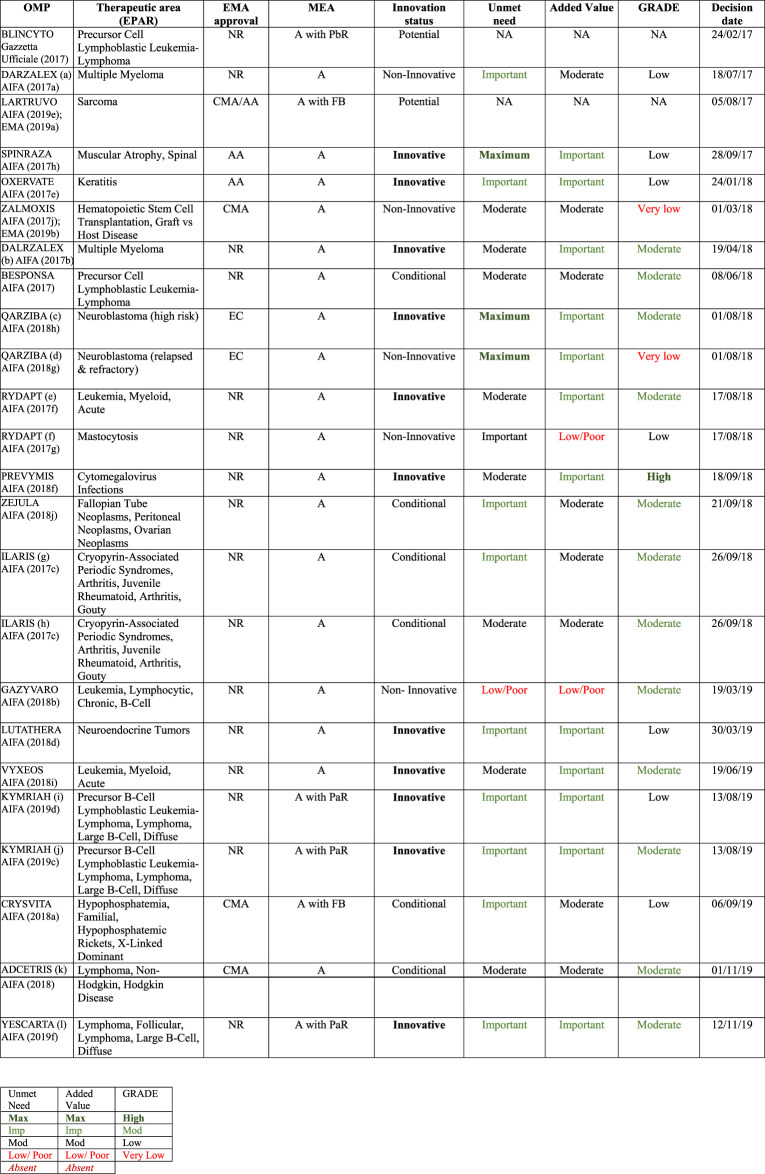

AA, accelerate assessment; CMA, conditional marketing authorisation; CTS, *Commissione Tecnico-Scientifica*; EC, exceptional circumstances; NR, normal route.

* Registry closed.

a For the treatment of relapsed and refractory multiple myeloma, whose previous therapies have included a proteasome inhibitor and an immunomodulator, and which have shown disease progression during the last therapy.

b For the indication in combination with lenalidomide and dexamethasone, or bortezomib and dexamethasone, for the treatment of adult patients with multiple myeloma who have received at least previous therapy.

c High-risk neuroblastoma in patients from 12 months of age who have previously undergone induction chemotherapy achieving at least a partial response, followed by myeloablative therapy and stem cell transplantation.

d Patients with history of relapsed or refractory neuroblastoma, with or without residual disease.

e Only in combination with standard induction chemotherapy with daunorubicin and cytarabine and consolidation with high-dose cytarabine for adult patients with newly diagnosed acute myeloid leukemia (AML) with positive FLT3 mutation.

f Monotherapy for the treatment of adult patients with aggressive systemic mastocytosis (ASM), systemic mastocytosis with associated haematological neoplasm (SM AHN), or mast cell leukaemia (MCL).

g Periodic autoinflammatory fever syndromes in adults, adolescents and children from 2 years of age: hyperimmunoglobulin D syndrome (HIDS)/mevalonate kinase deficiency (MKD) and Familial Mediterranean Fever (FMF).

h Periodic autoinflammatory fever syndromes in adults, adolescents and children from 2 years of age: Tumor necrosis factor receptor-associated periodic syndrome (TRAPS).

i Diffuse large B-cell lymphoma (DLBCL) in adults whose cancer has come back or did not respond after two or more previous treatments.

j B-cell acute lymphoblastic leukaemia (ALL), in children and young adults up to 25 years of age whose cancer did not respond to previous treatment, has come back two or more times, or has come back after a transplant of stem cells.

k CD30 positive cutaneous T-cell lymphoma (CTCL).

l Relapsed or refractory diffuse large B-cell lymphoma (DLBCL) and Primary mediastinal large B-cell lymphoma (PMBCL), after two or more lines of systemic therapy.

As shown in Figure 2 and specifically for OMPs in the next paragraph in Figure 4A, there has been a plateauing in use of new registries for MEA in the last 2 years, most likely due to the initiation of the new AIFA innovativeness’ recognition scheme (Gazzetta Ufficiale, 2017c; Gazzetta Ufficiale, 2017e).

### Relationship Between AIFA MEA Registry and Form of Regulatory Approval

As the EMA OMP legislation supports fast-tracked regulatory processes for OMPs, it was felt important to compare the type (MEA) of registry and form of the EMA marketing authorization in terms of: normal route (NR), accelerated assessment (AA), conditional marketing authorization (CMA), or exceptional circumstances (EC). The analysis of all MPs in the database shows that 78.5% were NR, 13.3% AA, and 8.3% CMA/EC. For OMPs, the more frequent use of fast-tracked procedures is apparent with 60.2% approved by NR, 21.6% by AA, and 18.2% by CMA/EC. For each of the three categories of OMP regulatory authorization within the centralized procedures, the pie charts in Figure 4B show the percentage of OMP registries. The five orphan-indications with exceptional circumstances approval (Gazzetta Ufficiale, 2011; Gazzetta ufficiale, 2014; Gazzetta Ufficiale, 2017b; Gazzetta Ufficiale, 2018) are appropriateness registries and were counted as CMA.

### AIFA Innovation and Registries (2017–2019)

An analysis was carried out comparing the CTS judgment on the recognition of innovation (Gazzetta Ufficiale, 2017c; Gazzetta Ufficiale, 2017e) for the registries initiated during the period 2017–2019. Of the 50 registries, 40 are cancer drugs (80%) and 20 are OMPs (16 cancer and 4 non-cancer). The details of the OMP registries, regulatory, and innovation status are shown in (Table 2). We also included the three indications of Ilaris^®^ (AIFA, 2017c) (canakinumab), given that the therapeutic indications involved a rare disease area (autoinflammatory periodic fever syndromes). So, in total, our observation included 23 OMP indication-based registries with CTS innovativeness judgments.

Table 2 shows that there was only one PbR OMP registry during this period. This was for Blincyto^®^ (Gazzetta Ufficiale, 2017), which was authorized under the normal regulatory route and received a status of potential innovation (innovation criteria not published). The new form of PaR (AIFA, 2019; AIFA, 2019a) registries was initiated with the CAR-T cell therapies, which were authorized *via* the normal regulatory route and were considered innovative in all their indications. There were also two registries with FB schemes for OMPs that had conditional (Crysvita®) and potential (Lartruvo®) innovation status assigned initially. For Lartruvo®, the regulatory post-authorization study (for sarcoma) did not confirm clinical benefit of Lartruvo (EMA, 2019a; EMA, 2019b). Consequently, the treatment was no longer authorized for this indication and the registry was closed (AIFA, 2019e).

This analysis shows that there has been a move away from OB-MEA for OMP, apart from using the new format of PaR for the highly complex CAR-T cell therapies.

## Discussion

This study reviewed the terms and implementation of AIFA registries, trends and correlations with regulatory approvals, and the innovation status determined at NHS reimbursement to understand how use of registries has evolved in Italy and what lessons can be learned, particularly for the case of OMPs. This analysis, for the first time, attempts to correlate the presence of a registry and the drug innovation assessment with the managed entry agreement implemented.

This discussion reflects on the evolution of AIFA registries, the essential features of AIFA registries that may provide learnings for other systems, recent evaluations of the registries, and specific issues related to use of registries and MEA for OMPs.

### Evolution of AIFA Registries

The history of AIFA registries can be characterized over four important phases as represented in Figure 2:

1.2005–2011: Italy was an early adopter in the generation of real-world data internationally establishing a national web-based platform governed by the Italian medicines authority. Monoclonal antibodies for cancer were most common and a risk-sharing approach with industry was the basis for of the first MEA.
2.2012: End of the first platform and transition to the new one in early 2013. Given the mandatory use of registries for reimbursement of NHS treatments, paper-based data collection was required by healthcare providers (HCPs) and regions during, and for a period after, the transition. At the date of our analysis, paper-based registries were still in use for eight registries (AIFA, 2020b).
3.2013–2017: Proliferation of registries and MEA (mainly PbR). The overall structure of registries was changed to be modular and give more regions management rights. From this time, further therapeutic areas were covered. AIFA started the first regular analysis of registries and consequently closed some of them as part of PR renegotiations with the support of registries aggregate reports and an advanced dashboard showing metrics and key performance indicators.
4.2017–ongoing: CTS innovativeness’ recognition is linked with the decision to implement an AIFA registry. The use of PbR registries has diminished with more use of appropriateness’ registries for innovative drugs with some implementation of combined FbA systems. A new outcome-based payment model for innovative advanced and high-priced treatments in the form of PaR (AIFA 2019; AIFA, 2019a) has been implemented. AIFA has started dissemination of the first registries’ analysis (AIFA, 2020b) on its web page as the result of the PR renegotiations.

The first three phases are primarily linked to the development of the registry system—launch of the concept of monitoring and a risk-sharing approach (phase one) and that of improving the web-based platform and greater active involvement of the various stakeholders (phase two and three). The fourth phase appears to be related to the policy change at AIFA with the launch of the recognition of innovativeness in 2017, optimizing the monitoring to focus registries on innovative drugs. To this is added the issue of simplification in general including that of avoiding the administrative burden associated with the monitoring process plus that of the implementation of the single MEA for single MP and single therapeutic indication. This would explain the disappearance of the patient-level MEAs implemented directly in the registries (with the exception of the new OBMEA payment model of the two Car-T cell therapies) leaving only that of the verification of appropriateness with the addition of eventual population-based financial agreements implemented outside the registries system.

### Essential Features of AIFA Registries

**FIGURE 5:**
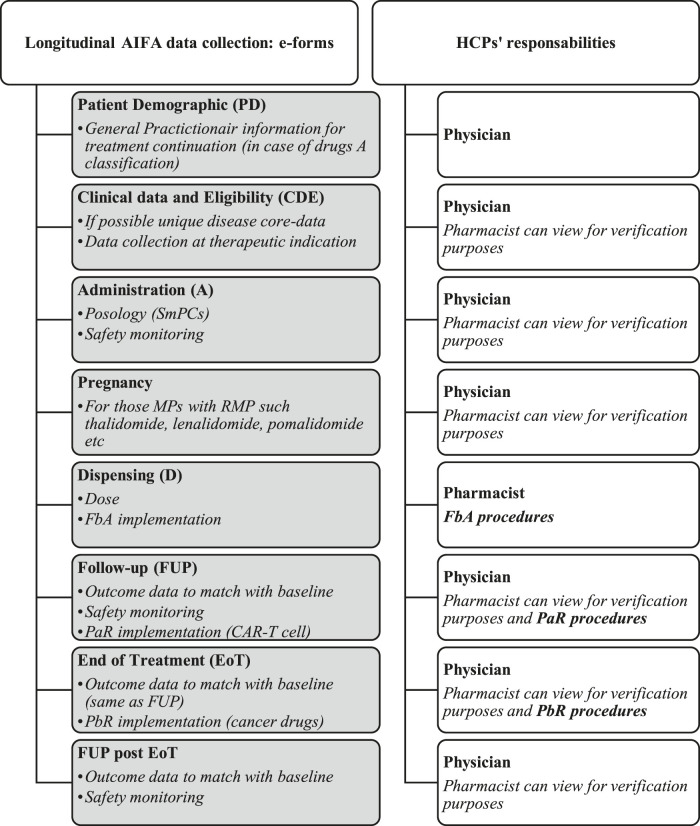
Stakeholder responsibilities within AIFA longitudinal registries. FbA, financial-based agreement; PaR, payment at result; PbR, payment by result.

The web-based system of AIFA registries was established to enable fair and equitable treatment of all patients in Italy, using a national system that is co-managed with the Italian regions to govern medicines’ use and expenditure. The registries have been designed to collect patient longitudinal data at public or private (NHS affiliates) hospitals and pharmacies, regions, district health services, and MAH. As reported in Figure 5, clinicians and the pharmacists are the main actors with both clinical and administrative responsibilities. There is no patient involvement at any stage in the development or analysis of AIFA registries.

The modular architecture of the registry platform that has been in place since 2013 can carry out automatic reporting and in-depth analyses, guaranteeing better data quality and reducing the time commitment of users. The three main features of the platform are:

1.Unique electronic patient demographic e-form enables linkage of records by patient across several MP registries. This allows analysis of populations treated with MP in dissimilar therapeutic areas (e.g., onco-hematology, osteoporosis, hypercholesterolemia, and arthritis) or following the lines of treatments for a disease (e.g., in cancer).
2.Distinct roles and responsibilities of stakeholders nationally and in regions. Regions need to support registries infrastructure, authorize prescribing centers, and certify HCPs.
3.Establishment of a core-disease data set—the registries are predominantly drug/indication-based, but in 2014 (AIFA, 2014a) a disease-based registry approach was started driven by regulatory discussions [adaptive pathway (Faulkner et al., 2016)], the initiative for patient registries (McGettigan et al., 2019), and interactions in different European working groups and projects (ADAPT SMART, 2015; EC, 2016; Bouvy et al., 2017; Moseley et al., 2020). The preference is to standardize data collection by disease (same core data) altering the patient eligibility based on the authorization characteristics of each product (as a result of the EMA label indication and AIFA restrictions).

These elements are shown in Supplementary Appendix 1, which is a representation of the inclusion and exclusion criteria in the Spinraza® appropriateness registry (AIFA, 2017i). This case shows the level of detail collected and treatment rules. All fields are mandatory, some of them contribute to determining eligibility for treatment and outcome-based follow-ups at pre-specified timepoints are required.

This structured approach to real-world data collection in MEA enabled by the AIFA registries can be contrasted with approaches taken in most other countries that curate data from a range of sources and are bespoke for individual products/indications. The standardized AIFA system overcomes the issues of data curation and enables systematization of approach, but perhaps as they are easier to establish, less consideration is given to defining when they should be used.

### Optimizing Use of Registries

Of the 283 AIFA indication-based registries established since 2005, only 77 have been closed. If an AIFA registry is running, HCPs must enter data into the AIFA registry system before they can prescribe it or obtain NHS reimbursement. Hence, the long duration of many of these registries has considerable administrative impacts in clinical practice.

One of the main objectives of the AIFA registries was to guarantee the sustainability and affordability of therapies, reducing the time of PR negotiation for innovative drugs by speeding up access to patients. MEAs reduce time to regional patient access for innovative and costly oncologic drugs (Russo et al., 2010), and OBMEA work in circumstances in which the payer is able to monitor patients over time and obtain refunds from industry if responses are not achieved in the real-world setting. These registries generate evidence from real-world clinical practice, representing an opportunity for collaboration among patients, academia, regulators, HTAs, payers, and industry to undertake analyses that can support learning health systems and improve patient outcomes (Russo et al., 2010; Facey et al., 2020). But still very little has been done on this front. Citing Garattini et al. (2015) “*it is clear that they* (drugs subjected to MEA and, more in general, under AIFA registries) *closely reflect the approval indication, hardly asking for any additional information useful for an extended clinical assessment. So, the information collected is unlikely to contribute to the existing evidence on the drugs under these agreements, beyond self-certified validation of appropriate prescription by the prescriber*.” Data access and confidentiality may be seen as a barrier, but experience has been gained in individual PR contracts between companies and AIFA and through the overarching memorandum of understanding between the pharmaceutical industries (Memorandum AIFA-Farmindustria, 2014). This document, created in 2014, could be updated and extended, inserting new rules that take into account the potential use of registries’ data and the specific needs of NHS (specially to avoid data duplication), the pharmaceutical industry, patient organizations, academia, and other scientific research. Furthermore, advances in data science (artificial intelligence, deep learning, and natural language processing) are generating a dynamism in processes that could be perceived as trying to disrupt human conventions (Car et al., 2019) and transform existing ways of working to enable a more contemporary approach that could meet many stakeholder expectations.

There is a need to start taking advantage of the immense data “lake” that has developed over the 14 years of AIFA registries. At the date of our analysis, AIFA had just started publishing analysis reports when registries have been closed or changed as a result of the MEA (AIFA, 2020). Up to April 2020, the first three published reports related to two MPs:

1. Abiraterone acetate (Zytiga®)
   ○ Appropriateness registry (A) with PbR for metastatic castration-resistant prostate cancer (mCRPC) during and post-chemo started on April 06, 2013, then transformed in A (PbR ended on July 26, 2017) and definitively closed on March 28, 2018 (AIFA, 2020e).
   ○ Appropriateness registry (A) with FbA [precisely a cost-sharing (CS)] for mCRPC pre-chemo started on September 30, 2014, then transformed in A (CS ended on July 26, 2017) and definitively closed on March 28, 2018 (AIFA, 2020f).
2. Enzalutamide (Xtandi^®^)
   ○ Appropriateness registry (A) with FbA (CS) for mCRPC post-docetaxel started on December 25, 2014, then transformed in A (CS ended on April 22, 2016) and definitively closed on September 07, 2018 (AIFA, 2020d).

These reports contain data collection information at the national and regional level on the number of treatments (ongoing, with end of treatment or discontinued), patient’s demographics, the number of treatment cycles, dispensing packages, and reason for treatment discontinuation. In terms of the MEAs’ implementation, it is interesting to note that there are only a few outstanding requests for the dispensing e-forms, which are obligatory for MEA implementation (national medians of 3.3%, 1.5 and 0.4%, respectively). This demonstrates that payback MEA are managed timeously within the registry system.

From these three published reports, we observe that: 1) the MEA can be modified without closing the registry; 2) the closure of indication-based registries for the same disease and for the same MP occurs at the same time; and 3) it is possible to apply different agreements for the same MP (indication-based pricing). Another reflection is regarding the monitoring duration: the three reports covered a minimum of 3.7 years and a maximum of 5 years. It would be interesting to see these types of reports for all the registries closed to date, especially for OMPs with longer durations (such as Nexavar® for renal cell carcinoma that ran for 12 years). Our analysis demonstrated that of the 77 registries already closed, there were eight registries with a duration of more than 10 years, with the maximum for ibritumomab tiuxetan at 14.05 years. For those still active—on the date of our analysis December 31st, 2019—there are 19 registries with a duration over 10 years (with a maximum of 15.75 years for bevacizumab), 73 registries have a duration between 5 and 9 years, and 41 registries have a duration between 3 and 5 years (including extremes).

The return on investment of AIFA registries is unclear, as the “return” is multifaceted and difficult to capture in full. The number of therapeutic failures (PbR) is important in terms of MEA impact, but AIFA has highlighted (AIFA, 2020a) that an assessment of efficacy should consider the value of appropriateness verification that is guaranteed by all the registries. This is impossible to measure. In its recent annual report (AIFA, 2020a), AIFA published aggregated reports for MP in some therapeutic areas of interest such as: chronic hepatitis C, age-related macular degeneration, family hypercholesterolemia (PCSK9) on the characteristics of the patients treated, and prescribing centers in the regions. Unfortunately, neither the clinical outcomes nor the form of the MEA were presented. Regarding the total MEA obtained in 2019 (AIFA, 2020a), 69% of the payback (€ 119,368,022) relates to financial agreements, with 44.8% for CS agreements and 24.2% for Capp agreements. The PbR and RS, cover 20.8% of paybacks, with RS representing a very small percentage (0.06%). The payback percentages by the ATC level are instead distributed mainly on two categories: 79.5% for antineoplastic and immunomodulatory drugs (L) and 18.9% for general antimicrobials for systemic use (J). Sense organ drugs (S) follow with 1.3%, nervous system drugs (N) with 0.2%, systemic hormone preparations, excluding sex hormones (H) and drugs of the muscular and skeletal system (M) with 0.04 and 0.0004%, respectively.

Furthermore, analysis of the AIFA registries could provide important scientific contributions in international peer-reviewed journals, such as that published recently for chronic myeloid leukemia (CML) (Breccia et al., 2020). That study analyzed the frequency of Italian patients who switched to a second-line therapy from a first line second-generation tyrosine kinase inhibitor such as dasatinib (Sprycel®) and nilotinib (Tasigna®), based on appropriateness registries with FbA for first line CML. However, it did not reflect on the value of the MEA or the implications of this analysis for the continuation of the MEA.

The literature about challenges with MEA in different jurisdictions is relatively large (Stafinski et al., 2010; Puig-Peiró et al., 2011; Wonder et al., 2012; Ferrario and Kanavos, 2013; Ferrario and Kanavos, 2015; Ferrario et al., 2017; Kanavos et al., 2017; Breccia et al., 2020; Zampiroli Dias et al., 2020) but there is a lack of published information about the factors that contribute to successful MEA systems and the constructs of MEA for individual products. Confidentiality clauses often drive the absence of published data in many countries (Stafinski et al., 2010; Wonder et al., 2012), however there is important information about the constructs of MEA that could be shared, including core data sets and aggregated analyses. Hence AIFA has an important opportunity to continue its new program of publishing reports of its closed registries and reflecting on the lessons learnt in the Italian context.

### AIFA Registries for Orphan Medicinal Products

Our analysis shows that almost 41% of the OMP registries are for the non-cancer area. From 2017, when the innovation assessment scheme was introduced, it appears that financial and OBMEA are no longer used and appropriateness registries are been preferred. The two CAR-T cell therapies with the new annual payment model [PaR (AIFA 2019; AIFA, 2019a)] are an exception.

As reported in Figure 4, for OMPs the use of fast-tracked regulatory procedures was used for 22.5% by AA, 12.4% by CMA, and 5.6% by EC. The trend of OMP registries are in line with the overall of MP trend with two interesting findings: the majority of OMPs are approved *via* normal regulatory processes (53/88) and most MEAs for OMPs are for appropriateness 72.7% (A), 15.9% A with PbR, 8.0% in A with FbA, and 3.4% in those with PaR.

### The Evaluation of Innovation

The focus on OMP registries and innovativeness’ recognition demonstrates how fundamental concepts such as therapeutic need, added clinical value, and the robustness of clinical evidence feature in NHS reimbursement. As shown in Table 1 and Table 2 the AIFA policy in recent years has been to clarify determination of innovation and require all products that achieve some form of innovative status to establish a registry. Even for OMPs, this has been the simplest form of appropriateness registry in most cases, requiring PR negotiation to obtain the best possible price without the complexity of an OBMEA applied in the registry. The balance between the three innovation criteria for the final CTS judgment is also interesting. The lack of association between levels achieved on the individual criterion and the overarching status shows that this is not a rigid scheme, but is considered in a wider context that takes account of the specificities of the case, especially if it is a rare disease (Fortinguerra et al., 2020).

Interestingly, there is one OMP (with an A registry) (letermovir, Prevymis® for prevention of cytomegalovirus) where the evidence is of high quality according to the GRADE method. For the other OMP registries, 12 had a moderate GRADE, seven low, and two very-low. It was envisioned that lower grade evidence might be apparent with OMPs and this might be linked to the form of registry, but no such pattern is clear to date. As reported from other authors (Fortinguerra et al., 2020; Galeone et al., 2021), the added value is the most dominant parameter. As highlighted in Table 2, the 11 OMPs recognized as innovative have an important score for the criterion of added value.

From a public health point of view, the level of innovation of a medicine is primarily defined by the benefit to patients. Benefits that have domains: therapeutic, clinical, or quality of life but also socioeconomic. Specially, the last criteria is not evident in the AIFA model, although the Agency covers not only a regulatory role but also that of payer based on an HTA methodology. It is not clear how the innovation model (added value ranking is not used in PR negotiations) will manage the various degrees of uncertainty which, although declined in the three criteria (therapeutic need, added value, and robustness of clinical evidence assessed with the GRADE method), are not easy in the interpretation of the final decision by AIFA.

### Limitations

The present study has some limitations. The analysis is based on registries in which the MP has obtained an NHS reimbursement in the Italian territory. This does not include medicines where the pharmaceutical company has not applied for PR submissions or those products that are assessed and assigned in C-classification (not reimbursed by NHS). We identified 88 OMP registries (13 are 648/96-R) for 52 OMPs. Different OMPs have more than one indication, with some in early access (brentuximab, ponatinib, pomalidomide, lenalidomide, eculizumab, and thalidomide). Our numbers are consistent with Villa et al. (2019) who reported 44 orphan drugs whose pharmaceutical companies have made a request for PR in Italy between 2013 and 2017 out of a total of 66 (11 still negotiating and other 11 not-reimbursed).

The analysis is limited to the information published on the AIFA registries webpage and cannot consider the various types of commercial MEA linked to the confidentiality of the agreement (PbR timing or discount percentage in the CS and the specific features of a Cap). In addition, the MEAs implemented outside the registry system are not covered in this study. Lastly, the analysis on innovativeness recognition and OMP registries is limited to the past 2 years based on publicly available reports.

## Conclusions

Although AIFA has the most well-established national registry system to support MEA, there are few articles regarding the characteristics of AIFA registries and none that focus on the specificities of OMPs. Evaluation of the evolution of the registry system over time in light of various regulatory and PR policy changes is informative, showing a move away from more complex outcomes-based schemes.

As more treatments come to market with an orphan status due to the advent of stratified medicine, mechanisms to deal with those products that have a high price and substantial uncertainties are needed. An OBMEA may be the solution, but no other country has such a structured system for data collection as Italy. Hence this exploration of the AIFA national registry system to support MEA can help policy makers in other countries understand the opportunities for, and feasibility of such a system, whilst considering challenges that may arise. In particular, the burden of data collection on the system needs to be balanced against the purpose of the MEA and the duration of data collection reviewed carefully. Other countries can balance the consideration of investment in a national web-based system that allows bespoke data collection for each treatment indication vs. curation of real-world data from their own health system. This balance will differ for each country depending on their data infrastructures (with some countries having high quality, accessible electronic health records).

This analysis has shown that AIFA has used MEA for most of the MPs that it has reimbursed. Klemp et al. (2011) and Nguengang Wakap et al. (2020) state that MEA may be difficult to negotiate, require legal input, increase bureaucracy, and should specify methods of review and termination. AIFA has overcome many of these obstacles through national web-based data collection systems that are mandatory to allow manufacturer reimbursement, clinicians to prescribe, and pharmacists to dispense. This has been driven within a legal framework and required investment in a data platform. But several critical points on registries, MEAs, and the huge national efforts and investments in data collections still remain unanswered: what is the return in terms of additional evidence generation? Why do some MEA have such long duration and what knowledge is gained in such long periods, what is the decision-support of these long MEA? How the use of registries and MEAs has generated positive impact on the sustainability of the healthcare system providing a real support for value-based pricing? It is clear that the data collected and the MEAs are part of the regular PR renegotiations, but no results on MEA’s efficiency and performance have been published so far given the confidentiality of the agreements present. In addition, there are no cases of stopping of reimbursement.

AIFA has established a registry system that ensures all patients receiving treatment are eligible to initially receive, and continue on, the treatment. The system can also be used to administer individual-based payback/payment schemes based on the outcomes of individual patients. Given the quality of the data and long duration of data collection, there is potential to aggregate data across patients and undertake analyses that seek to determine optimal use of the treatment and create a learning health system. Furthermore, there is a need for greater transparency with the analyses, and for this, new agreements with marketing authorization holders may be needed that allow analyses to be published.

This research has been used alongside the other research from the IMPACT HTA project to document learning about the initiation and implementation of OBMEA for OMPs. A case study analysis of OBMEA across countries in the EU and Australia, concluded that OBMEA should be “the exception and not the rule,” with concerns about the burden placed on all stakeholders involved (Facey et al., 2021). Those case studies also highlighted the need for better alignment of data collection requirements across health jurisdictions and collaboration on approaches that could enable aggregated data sharing. Given AIFA’s wealth of experience and data, there is an opportunity for Italy to take a lead in this study.
