# Supplementary material for: The Evolution of AIFA Registries to Support Managed Entry Agreements for Orphan Medicinal Products in Italy
Source: Front Pharmacol. 2021 Aug 10;12:699466. doi: 10.3389/fphar.2021.699466 (PMC8386173; doi:10.3389/fphar.2021.699466)
Supplement: Supplementary file 1 [file Image1.pdf]

1. Clinical & eligibility data

Patient with a genetically confirmed diagnosis of SMA 5q (mutations in the SMN1 gene)[1]

Yes ○ No ○

Availability of the SMN2 gene copy number

Yes ○ No ○

SMN2 gene copy number

Yes <=4 No

Presumed SMA phenotype (TYPE)

TYPE I: Symptom onset occurs within the first 6 months of life (never sitting achievement)

TYPE II: Symptom onset between 7-18 months with sitting achievement (although in the past and currently lost)

TYPE IIIA: Symptom onset between 18 months and before the 3rd year of age with sitting achievement (although in the past and currently lost)

TYPE IIIB: Symptom onset after 3 years of age

0 ○ I ○ II ○ IIIA ○ IIIB ○ IV ○

Indicate if the forced vital capacity measurement (FVC) has been performed

Yes ○ No ○

FVC Value

Mobility & physical test: indicate one of the tree tests and complete

- 0-4 for each 16 item ○ CHOP INTEND
- 8 items ○ Motor milestones HINE, sez. 2 Milestone level progression and age (2-24 months) expected in health infants
- 0-2 for each 33 item ○ HFMSE

Disease onset date

_ / ___ / ___

Diagnosis date

_ / ___ / ___

Symptomatic patient

Yes ○ No ○

Patient age at time of diagnosis

Check control with patient birth (as indicated in demographic form) and diagnosis date

Patient able to maintain a stable

Yes ○ No ○ NA ○

Ambulatory patient?

Yes ○ No ○ NA ○

Presence of respiratory complications?

Yes ○ No ○

The patient is in assisted ventilation?

Yes ○ No ○

Patient already in treatment with Spinraza AND in agreement with AIFA registry criteria[2]?

Yes ○ No ○

Number of drug administrations

Start treatment date

_ / ___ / ___

1. For the presumed SMA phenotypes, refer to:
Arnold WD, Kassar D, Kissel JT. Spinal muscular atrophy: diagnosis and management in a new therapeutic era. *Muscle Nerve*. 2015;51(2):157-67.

Mercuri E, Bertini E, Iannaccone ST. Childhood spinal muscular atrophy: controversies and challenges. *Lancet Neurol.* 2012;11(5):443-52.

2. Italian Early Access regulations as Ministerial Decree of 18 May 2017, 648/1996 Law, 326/2003 Law (5% Fund) or 94/1998 Law (Di Bella)

2. Administration

Administration date

__/__/__

Administration number

Posology

12 mg (5 ml) ○

Refer to the SmPC, paragraph 4.2

The duration of administration 1 and 2 is 14 days. The duration of administration 3 is 35 days. The duration of subsequent administrations (after the third) is 120 days.

Dose

Loading ○ Maintenance ○

Day 0

Day 14

Day 28

Day 63

Every 4 months

n

Have the contraindications, special warnings and precaution for use reported in paragraph 4.3 and 4.4 of the SmPC been checked (verified) by the physician?

Yes ○ No ○

Indicate if there have been adverse drug reactions to the previous administration

Yes ○ No ○

Link to AIFA Pharmacovigilance network

3. Dispensing

Dispensing date

__/__/__

Dispensing number

SPINRAZA

Marketing Authorisation 045426018: 5 ml vial containing nusinersen sodium equivalent to 12 mg of nusinersen

N.

4. Follow-up

Follow-up date

__/__/__

Patient disease status

○ Improved
○ Worsened
○ Stable

Mobility & physical test: indicate one of the tree tests and complete

○ CHOP INTEND
○ Motor milestones HINE, sez. 2 Milestone level progression and age (2-24 months) expected in health infants
○ HFMSE

Mandatory: FUP1 after the end of loading doses; subsequent FUP after each prescription of maintenance doses

5. End of treatment

End of treatment date

__/__/__

Mobility & physical test: indicate one of

○ CHOP INTEND
○ Motor milestones HINE, sez. 2 Milestone level progression and
○ HFMSE

End of treatment causes

○ Parents/caregiver/patient decision
○ Disease progression
○ Clinician decision
○ Lost to follow-up
○ Serious Adverse Event (SAE)
○ Complications related to the administration procedure
○ Inability to perform the administration procedure
○ Patient death

Indicate the death causes

○ Disease progression
○ Drug-related toxicity
○ Other: specify (free text)

Death date

__/__/__

Link to AIFA Pharmacovigilance network

6. Follow-up after end of treatment

Follow-up date

__/__/__

Patient disease status

○ Improved
○ Worsened
○ Stable

Mobility & physical test: indicate one of the tree tests and complete

○ CHOP INTEND
○ Motor milestones HINE, sez. 2 Milestone level progression and age (2-24 months) expected in health infants
○ HFMSE

Check-control with AIFA Deliberation date & other dates present at longitudinal patient data collection

Physician

Pharmacist

Not eligible

Eligible

Mandatory for eligibility

Mandatory

Treatment under early access regulation
